# Unveiling the Hidden Reservoir: High Prevalence of Occult Hepatitis B and Associated Surface Gene Mutations in a Healthy Vietnamese Adult Cohort

**DOI:** 10.3390/microorganisms14010238

**Published:** 2026-01-20

**Authors:** Huynh Hoang Khanh Thu, Yulia V. Ostankova, Alexander N. Shchemelev, Elena N. Serikova, Vladimir S. Davydenko, Tran Ton, Truong Thi Xuan Lien, Edward S. Ramsay, Areg A. Totolian

**Affiliations:** 1 Pasteur Institute in Ho Chi Minh City, Ho Chi Minh City 70000, Vietnam; thuhhk@pasteurhcm.edu.vn (H.H.K.T.); tont@pasteurhcm.edu.vn (T.T.); 2 Saint Petersburg Pasteur Institute, St. Petersburg 197101, Russia; shenna1@yandex.ru (Y.V.O.); genista.bio@gmail.com (E.N.S.); vladimir_david@mail.ru (V.S.D.); totolian@spbraaci.ru (A.A.T.); 3 Faculty of Pharmacy, Van Lang University, Ho Chi Minh City 70000, Vietnam; lien.ttx@vlu.edu.vn

**Keywords:** hepatitis B virus (HBV), chronic HBV infection (CHB), HBsAg-positivity, occult HBV infection (OBI), escape mutations, Vietnam

## Abstract

Vietnam faces a hyperendemic burden of hepatitis B virus (HBV) infection, but the prevalence of occult HBV infection (OBI) and its underlying molecular mechanisms in healthy populations remain poorly understood. This study aimed to characterize the serological and molecular HBV profile of a healthy Vietnamese adult cohort in Southern Vietnam. We assessed the prevalence of occult HBV infection (OBI) and HBsAg-positivity (serving as a proxy for probable chronic infection). In this cross-sectional study, 397 healthy adults from Southern Vietnam underwent serological screening for HBsAg, anti-HBs, and anti-HBc. All participants were screened for HBV DNA using a high-sensitivity PCR assay (LOD ≥ 5 IU/mL). For all viremic cases, the full Pre-S/S region was sequenced to determine genotype and characterize escape mutations. We uncovered a high prevalence of both HBsAg-positivity (17.6%) and OBI (9.3% HBsAg-negative, HBV DNA-positive). Serological analysis revealed a massive, age-dependent reservoir of past exposure (63.7% anti-HBc) characterized by a high and increasing prevalence of the anti-HBc only profile (31.5%), a key serological marker for OBI. This trend contrasted sharply with a steep age-related decline in protective anti-HBs. The viral landscape was dominated by genotypes B (73.8%) and C (26.2%), with sub-genotypes B4 and C1 being the most prevalent. Critically, individuals with OBI carried a significantly higher burden of S gene escape mutations compared to those with HBsAg-positivity (*p* < 0.001). Canonical escape variants, including sG145R (21.6%), sK141R/T/E/Q (24.3%), and sT116N/A/I/S (18.9%), were exclusively or highly enriched in the OBI group. A LASSO-logistic model based on this mutational profile successfully predicted occult infection with high accuracy (AUC = 0.83). A substantial hidden reservoir of occult HBV infection exists within the healthy adult population of Vietnam, driven by a high burden of S gene escape mutations. These findings highlight the significant limitations of conventional HBsAg-only screening. They also underscore the need for comprehensive molecular surveillance to address the true scope of HBV viremia, hopefully enabling a reduction in hidden transmission of clinically significant viral variants.

## 1. Introduction

The hepatitis B virus (HBV) remains a formidable global public health challenge and is a major cause of mortality and morbidity worldwide. Current estimates indicate that approximately 296 million people live with chronic HBV infection, leading to nearly one million deaths annually in recent years. This figure underscores the virus’s persistent impact through complications such as cirrhosis and hepatocellular carcinoma (HCC) [1,2]. The serological marker hepatitis B surface antigen (HBsAg) has been the cornerstone for diagnosing chronic infection for decades. Its prevalence is used to define geographic endemicity. Regions like the western Pacific, including Vietnam, have been historically classified as hyperendemic, with HBsAg prevalence ranging from 8% to as high as 25% in certain populations [3,4]. The introduction of universal infant vaccination in Vietnam in 2003 represents a monumental public health achievement, successfully reducing the incidence of new chronic infections among younger generations and gradually altering the country’s epidemiological profile [5,6,7]. However, this success has created a new challenge. The HBV burden is now increasingly concentrated in the older, pre-vaccination era adult cohort, who often carry the virus unknowingly. This group faces a lifelong risk of severe liver disease and constitutes a significant reservoir for ongoing transmission.

While HBsAg screening provides a critical window into the overt HBV epidemic, its reign as the definitive sentinel of infection has been fundamentally challenged by the recognition of occult hepatitis B infection (OBI). This condition is characterized by the presence of HBV DNA in the liver or serum of individuals who test negative for HBsAg, representing a hidden viral reservoir that eludes standard diagnostic surveillance [8].

HBV is predominantly transmitted through perinatal exposure, sexual contact, and close household contact worldwide. However, the clinical and public health implications of OBI are particularly pronounced in settings that rely on HBsAg-based screening, such as blood transfusion and organ transplantation, where low-level viremia may go undetected and result in inadvertent transmission. In addition, OBI can reactivate under conditions of immunosuppression (such as chemotherapy), leading to severe or fulminant hepatitis. It is also increasingly associated with silent progression of liver fibrosis and hepatocarcinogenesis, contributing to hepatocellular carcinoma development [8,9,10]. However, determining the true prevalence of OBI is challenging due to methodological heterogeneity in HBV DNA assays and considerable diversity among the studied cohorts [8,11,12].

The molecular mechanisms underpinning OBI are intricately linked to the complex interplay between the host immune system and the sophisticated viral mechanisms for persistence. A key factor is the potent suppression of viral replication and gene expression by the host immune response, which limits the virus to levels below the detection threshold of conventional HBsAg assays. However, this immune control is often incomplete and fails to eradicate the viral covalently closed circular DNA (cccDNA) minichromosome from hepatocyte nuclei. This creates a latent reservoir poised for potential reactivation. Compounding this host-driven mechanism is the remarkable genetic plasticity of HBV itself. The error-prone reverse transcriptase of its replication cycle fosters a diverse quasispecies population within a single host, enabling the selection of viral mutants adept at evading immune and diagnostic detection. Substitutions within the major hydrophilic region (MHR) can alter the antigenic structure of HBsAg [13,14]. Specifically, these include the immunodominant “a” determinant (aa 124–147), most notably the sG145R variant, and other key substitutions (sP120, sT126, sM133). These escape mutations can allow the virus to evade neutralization by vaccine-induced antibodies and, critically, prevent its detection by diagnostics, leading to false-negative HBsAg results [15,16].

This mutational landscape is further shaped by the global distribution of distinct HBV genotypes (A-J), which exhibit varying clinical outcomes, seroconversion patterns, and predispositions to specific mutations. In Vietnam, the viral ecosystem is characterized by the co-dominance of genotypes B/C, with sub-genotypes B4 and C1 being the most prevalent lineages. Genotype C is often associated with more aggressive liver disease [14,17,18,19]. A critical, and often overlooked, dimension of viral persistence arises from the complete genomic overlap of the S gene with the reverse transcriptase (RT) domain of the polymerase (P) gene. This architectural feature means that selection pressure from nucleos(t)ide analog therapy, a cornerstone of chronic HBV management, can have unintended consequences. For instance, the well-documented adefovir-resistance mutation rtA181T frequently co-emerges with the sW172* stop codon in the overlapping S gene. This results in truncated, secretion-defective surface proteins that can lead to HBsAg serological clearance despite ongoing viremia, effectively creating an iatrogenic form of OBI [20,21,22,23].

In the contemporary Vietnamese context, studies simultaneously integrating comprehensive serological profiling, HBV DNA detection, and viral genetic analysis in apparently healthy adult populations are still limited. As a result, key questions remain regarding the regional burden of OBI, its molecular correlates, and its contribution to the hidden HBV reservoir in Southern Vietnam. Our study’s focus on a healthy adult population aligns directly with key strategic recommendations from both the World Health Organization (WHO) and the Vietnamese Ministry of Health. The WHO advocates for universal HBV screening for all adults in settings with an HBsAg prevalence of ≥2%, while encouraging the integration of testing into routine medical services to identify the vast number of undiagnosed individuals [24]. Similarly, Vietnam’s national guidelines specifically identify individuals undergoing general health check-ups as a priority group for screening [25]. Therefore, this study aimed to characterize the serological and molecular HBV profile of a healthy Vietnamese adult cohort in Southern Vietnam. We assessed the prevalence of occult HBV infection (OBI) and HBsAg-positivity (serving as a proxy for probable chronic infection). We also sought to identify viral escape mutations associated with occult infection, thereby elucidating the hidden viral reservoir missing by conventional screening.

## 2. Materials and Methods

### 2.1. Materials

A total of 397 adults attending routine examinations for non-medical reasons at healthcare facilities in Southern Vietnam were enrolled. “Non-medical reasons” refers to presentations for general health checks or employer/visa screening. This pragmatic sample was selected to capture an ostensibly healthy, healthcare-engaged population, in line with WHO and Vietnamese Ministry of Health screening priorities. All participants were informed about the study (objectives, methodology) and provided with written informed consent. De-identified medical records provided information on age, chronic conditions, and blood-borne infections. This study was conducted within the framework of a formally approved scientific collaboration under a memorandum of understanding (signed 15 December 2017) between the Pasteur Institute in Ho Chi Minh City (Vietnam) and the Saint Petersburg Pasteur Institute (Russia). Ethics approvals were obtained from: the Saint Petersburg Pasteur Institute Ethics Committee (protocol No. 47, 25 December 2018; protocol No. 92, 29 October 2019); and the Pasteur Institute in Ho Chi Minh City Ethics Committee (protocol No. 36/GCN-PAS, 16 December 2019). Exclusion criteria included: a history of HIV infection, tuberculosis, or viral hepatitis (B, C, D).

Among 397 conditionally healthy individuals attending for non-medical reasons, the gender distribution was balanced: 49.1% men (95% CI: 44.2–54.0) and 50.9% women (95% CI: 45.9–55.9). Participants were aged 18–65 years with a median age of 36.0 (IQR 28.0–45.0). They were predominantly younger than 50 years old, including those: <30 (*n* = 119, 30.0%); 30–39 (*n* = 118, 29.7%); and 40–49 (*n* = 108, 27.2%). Those aged 50–59 (*n* = 35, 8.8%) and ≥60 (*n* = 17, 4.3%) were relatively less represented.

### 2.2. Methods

#### 2.2.1. Sample Transportation and Storage

For each participant, 10 mL of venous blood was drawn from the antecubital vein into BD Vacutainer^®^ K2-EDTA tubes (Becton Dickinson Medical (S) Pte. Ltd., Singapore). All samples were subsequently de-identified to protect patient privacy. Plasma was isolated by centrifugation (1500× *g*, 20 min, 4 °C) (Beckman Coulter, Inc., USA). To enrich viral particles in low-level viremic cases, all samples subsequently underwent ultracentrifugation (24,000× *g*, 1 h, 4 °C). Finally, concentrated plasma was aliquoted into cryovials for downstream serological testing (500 µL), molecular screening (500 µL), and sequencing (5 mL) applications. Samples were transported within 24 h of collection using specialized biomaterial containers maintained at +4 to +8 °C.

#### 2.2.2. Enzyme-Linked Immunosorbent Assay

ELISA analysis for HBV markers involved the qualitative determination of HBsAg, anti-HBs IgG, and anti-HBc IgG as previously described [26,27]. All tests were performed in duplicate according to manufacturer instructions using the following reagent kits: DS-EIA-HBsAg, DS-EIA-ANTI-HBs, and DS-EIA-ANTI-HBc (Diagnostic Systems RPC, Nizhny Novgorod, Russia); as well as Vectohep B-HBs-antigen, VectoHBsAg-antibodies, and HepaBest anti-HBc-IgG (Vector-Best, Novosibirsk, Russia). The HBsAg assay was performed using a reagent kit with a sensitivity of 0.01 IU/mL [28,29].

#### 2.2.3. Nucleic Acid Extraction

Initial HBV DNA extraction for PCR screening was performed on 500 µL of plasma using the RIBO-prep commercial kit (AmpliSens, Moscow, Russia). For samples that tested positive for HBV DNA, a subsequent large-volume extraction was conducted using ≥5 mL of plasma to obtain sufficient material for sequencing. This second extraction utilized the NK-Magno-UltraPure-A reagent kit (NPF Epitop LLC, Saint-Petersburg, Russia) following the manufacturer’s protocol with modifications to accommodate the larger sample volume.

#### 2.2.4. HBV DNA Amplification and Sequencing

Our multi-step molecular detection strategy was designed to maximize the identification of low-level viremia in accordance with the recommendations of the Taormina Workshop on Occult HBV Infection. Initial screening was performed using the “AmpliSens^®^ HBV-FL real-time PCR” commercial kit (AmpliSens, Moscow, Russia), which has an analytical sensitivity of 50 IU/mL. To ensure the detection of all low-viremic cases, samples were subsequently analyzed with a highly sensitive in-house method specifically optimized for HBsAg-negative (5 IU/mL lower limit of detection) [30,31]. For all samples confirmed to be HBV DNA-positive, the Pre-S1, Pre-S2, and S regions were then amplified for sequencing using a nested PCR protocol with a set of flanking primers [11,12,32].

#### 2.2.5. HBV Genotype and Mutation Analysis

Following sequencing, raw fragment data were assembled into consensus nucleotide sequences using Unipro UGENE software v.51 [33]. Assembled sequences were initially identified using the BLASTn algorithm against the GenBank database; they were subsequently deposited therein. For phylogenetic analysis, sequences were aligned using the ClustalW algorithm in MEGA12 [34], and a maximum likelihood tree was constructed with 1000 bootstrap replicates to assess branch support. To identify clinically significant variants, nucleotide sequences were translated into amino acid sequences and analyzed for mutations using the HBVseq, HBVdb, and Geno2pheno online databases [35,36,37].

#### 2.2.6. Statistical Analysis

All statistical analyses were performed using STATA version 17 (StataCorp LLC, College Station, TX, USA). A two-sided *p*-value < 0.05 was considered statistically significant. Categorical variables were described using frequencies and percentages, with 95% confidence intervals (CI) calculated for proportions. Differences in proportions between groups were assessed using the Chi-square (χ^2^) test or Fisher’s exact test, as appropriate. The Wilcoxon rank-sum (Mann–Whitney) test was used to compare the non-normally distributed counts of mutations per patient between the HBsAg-positive and OBI groups.

Multivariable logistic regression models were used to calculate odds ratios (OR) and their corresponding 95% CIs to identify independent predictors for various serological and virological outcomes. To evaluate the predictive power of escape mutations for OBI, a penalized logistic regression model with the least absolute shrinkage and selection operator (LASSO) was developed. The performance of this model was assessed by calculating the area under the receiver operating characteristic curve (AUC).

## 3. Results

### 3.1. Prevalence and Distribution of HBV Serological Markers

In the study population, 16.12% of participants were completely seronegative for all three HBV markers. Evidence of previous HBV exposure without detectable HBsAg was the most common, with ‘anti-HBc IgG only’ positivity observed in 31.49%, and combined anti-HBc IgG with anti-HBs IgG positivity in 17.13%. ‘Anti-HBs IgG only’ positivity, suggesting vaccine-induced immunity, was detected in 17.63%. HBsAg-containing profiles accounted for 17.63% of participants. The predominant HBsAg-positive profile was concurrent HBsAg and anti-HBc IgG positivity (11.59%), indicating active infection with evidence of past exposure. Less frequent profiles included HBsAg only positivity (2.23%), triple-marker positivity (HBsAg, anti-HBc IgG, anti-HBs IgG; 3.52%), and the rare coexistence of HBsAg and anti-HBs IgG without anti-HBc IgG (0.25%). Detailed serological distributions are shown in Table 1. Significant gender differences were observed across several HBV markers. The prevalence of HBsAg was higher in men than in women (χ^2^ = 14.83, *p* < 0.0001), with men showing 2.89-fold increased odds of HBsAg positivity (95% CI: 1.61–5.31). Similarly, anti-HBc IgG positivity was more common in men (χ^2^ = 8.22, *p* = 0.0041, OR = 1.83, 95% CI: 1.18–2.84). In contrast, ‘anti-HBs IgG only’ was significantly less prevalent in men (χ^2^ = 6.11, *p* = 0.0134, OR = 0.51, 95% CI: 0.29–0.90).

### 3.2. Prevalence of HBV DNA Positivity and Occult HBV Infection

Among the healthy individuals tested, 26.95% (95% CI: 22.65–31.5) were HBV DNA positive, of whom 17.63% (95% CI: 14.01–21.74) were concurrently HBsAg positive. The overall prevalence of occult HBV infection was 9.32% (95% CI: 6.65–12.62) among 397 adults. OBI prevalence was comparable between males (9.23%, 95% CI: 5.56–14.20) and females (9.41%, 95% CI: 5.76–14.30). Among participants with detectable HBV DNA and HBsAg positivity (*n* = 70), males accounted for 70.0% (95% CI: 57.87–80.38), whereas females represented 30.0% (95% CI: 19.62–42.13). In contrast, among HBV DNA-positive but HBsAg-negative individuals (*n* = 37), percentages were similar between males (48.65%, 95% CI: 31.92–65.60) and females (51.35%, 95% CI: 34.40–68.08) (Table 2). In the logistic regression model, the HBV DNA^+^/HBsAg^+^ profile showed a significant gender difference, with men having 2.89-fold higher odds of viremic HBsAg-positive status compared to women (χ^2^ = 14.83, *p* < 0.0001, 95% CI: 1.61–5.31). In contrast, the model indicated no significant gender difference in occult HBV infection (χ^2^ = 0.00, *p* = 0.952, OR = 0.98, 95% CI: 0.47–2.04), suggesting that gender was not a predictor of this serological profile.

### 3.3. Integrated Analysis of Age-Related Trends in Serological and Molecular Markers

**Figure 1:**
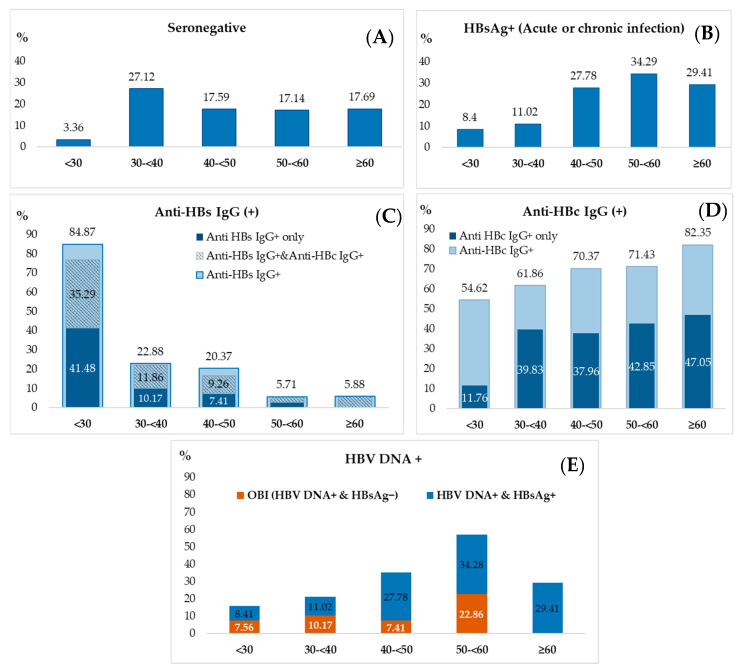
Serological marker prevalence by age group. Percentages indicate the prevalence of each specific marker/profile within the age group featuring the following sample sizes: <30 (*n* = 119); 30–39 (*n* = 118); 40–49 (*n* = 108); 50–59 (*n* = 35); ≥60 (*n* = 17). (**A**) Seronegative. (**B**) HBsAg positivity. (**C**) Anti-HBs IgG positivity. The full bar height represents anti-HBs IgG positivity, composed of: ‘anti-HBs IgG^+^ only’ (dark blue); anti-HBs IgG combined with anti-HBc IgG (patterned); and anti-HBs IgG combined with other markers (light blue). (**D**) Anti-HBc IgG positivity. The full bar height represents anti-HBc IgG^+^, composed of: “anti-HBc IgG^+^ only’ (dark blue) and anti-HBc IgG combined with other markers (light blue). (**E**) HBV DNA positivity subdivided into HBsAg-positivity (blue) and occult hepatitis B infection (HBsAg-negative/OBI, orange).

The prevalence of serological and molecular markers across different age groups is presented in Figure 1. Age-specific distributions of HBV serological and molecular markers are shown in Figure 1. Logistic regression demonstrated a significant association between age and HBsAg positivity (χ^2^ = 26.07, *p* < 0.0001, df = 4). Compared with individuals younger than 30 years, the odds of HBsAg positivity were significantly higher in all age groups ≥40 years, whereas no significant difference was observed in the 30–39 year group. Pairwise comparisons indicated that HBsAg prevalence increased markedly after the age of 40 and remained relatively stable thereafter.

A strong inverse association was observed between age and anti-HBs IgG positivity (χ^2^ = 169.13, *p* < 0.0001, df = 4), including the “anti-HBs IgG only” profile (χ^2^ = 58.11, *p* < 0.0001, df = 4). Relative to the <30 year reference group, the odds of anti-HBs IgG positivity declined sharply after the age of 30, with no consistent differences among adjacent older age groups.

Age was also significantly associated with anti-HBc IgG positivity (χ^2^ = 10.23, *p* = 0.0367, df = 4), with higher odds in older age groups compared with individuals under 30 years. This association was more pronounced for the “anti-HBc IgG only” profile (χ^2^ = 35.03, *p* < 0.0001, df = 4), which showed progressively increased odds across all age groups ≥30 years, without significant differences between adjacent categories.

In contrast, the odds of resolved HBV infection, defined as concurrent anti-HBc IgG and anti-HBs IgG positivity, declined significantly with age (χ^2^ = 52.01, *p* < 0.0001, df = 4). Younger individuals (<30 years) had the highest likelihood of this profile, while prevalence remained low and relatively stable in older age groups.

Finally, age was a significant predictor of the HBV DNA–positive/HBsAg-positive profile, indicative of active chronic infection (χ^2^ = 26.07, *p* < 0.0001, df = 4). The odds increased markedly after the age of 40 and plateaued in subsequent decades. In multivariable analyses, both age and sex were independent predictors of HBsAg, anti-HBs IgG, and anti-HBc IgG positivity.

To synthesize the observed age-dependent patterns, and quantify trends across key serological and viremic markers, we performed an analysis treating age as an ordinal variable. Table 3 reports adjusted odds ratios (aOR) per one-step increase in age group. Age was positively associated with HBsAg^+^ (aOR = 1.74, 95% CI: 1.37–2.21), HBV DNA^+^ (aOR = 1.59, 95% CI: 1.30–1.96), overall anti-HBc IgG^+^ (aOR = 1.37, 95% CI: 1.12–1.66), and the ‘anti-HBc IgG^+^ only’ profile (aOR = 1.55, 95% CI: 1.28–1.89). In contrast, age was negatively associated with overall anti-HBs IgG^+^ (aOR = 0.22, 95% CI: 0.16–0.31), ‘anti-HBs IgG^+^ only’ (aOR = 0.28, 95% CI: 0.19–0.42), and resolved infection (anti-HBc IgG^+^/anti-HBs IgG^+^, aOR = 0.42, 95% CI: 0.30–0.58). All trends were significant (*p* < 0.001 for all except overall anti-HBc IgG^+^, *p* = 0.0021). These estimates provide a unified quantitative summary indicating increasing chronic/viremic/exposure markers with advancing age, alongside declining immunity/resolution.

### 3.4. Serological Profiles Among HBV DNA–Positive Individuals

Among 107 HBV DNA–positive cases, 65.42% (95% CI: 55.84–73.90) were HBsAg^+^, and 34.58% (95% CI: 25.67–43.59) were OBI (HBsAg–). Profile distributions differed between the HBsAg-positive and OBI groups. In the HBsAg-positive group, the predominant profile was anti-HBc IgG positive without anti-HBs IgG (65.71%), whereas among OBI cases, this profile was less frequent (51.35%). In contrast, combined anti-HBc IgG and anti-HBs IgG positivity was more common in the OBI group (32.43%) than in the HBsAg-positive cases (20.00%). ‘Anti-HBs IgG only’ positivity was observed in 10.81% of OBI cases. This emphasizes the diagnostic gap when screening relies on HBsAg alone. The detailed distribution of serological profiles is presented in Table 4.

### 3.5. HBV Genotyping

**Figure 2:**
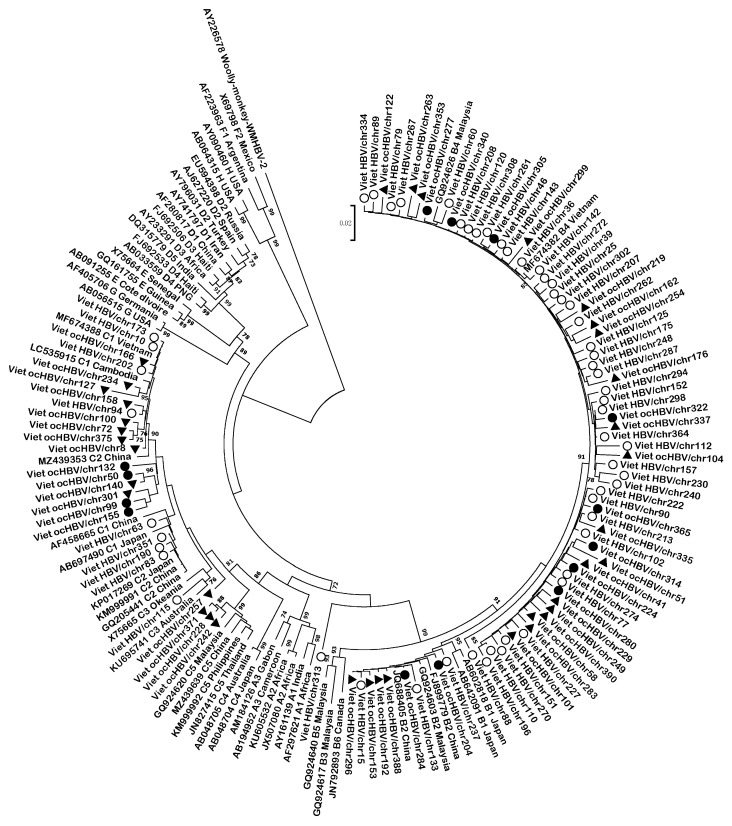
Phylogenetic analysis of HBV nucleotide sequences from conditionally healthy Vietnamese individuals. Study isolates are marked as open circles (○, HBsAg-positive), filled circles (●, initially OBI, HBsAg-positive by high-sensitivity assay), or filled triangles (▲, HBsAg-negative). They are shown alongside reference GenBank sequences (labeled by accession number, genotype, and origin). The tree is rooted with the Woolly Monkey HBV sequence (AY226578) as an outgroup. Bootstrap values >70% (1000 replicates) are displayed at the nodes.

Viral DNA was extracted from all 107 HBV DNA^+^ volunteer samples. The pre-S1/pre-S2/S region was sequenced to determine genotypes and to characterize amino acid substitutions in the polymerase reverse-transcriptase (RT) domain and in the surface proteins (SHB, MHB, LHB) of both HBsAg-negative and HBsAg-positive isolates. The pre-S1, pre-S2, and S region nucleotide sequences were submitted to GenBank (acc. numbers MZ671234–MZ671251, PX396142–PX396210). Phylogenetic relationships were inferred from these sequences (Figure 2).

**Figure 3:**
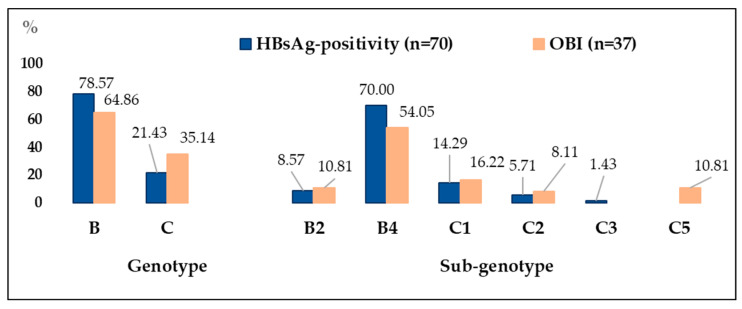
Distribution of HBV genotypes and sub-genotypes among individuals with HBV DNA by subgroup (HBsAg-positivity, OBI). Percentages indicate the relative representation of each genotype and sub-genotype within each group.

Among the 107 HBV DNA-positive cases, comprehensive genotyping and subtyping analysis, incorporating phylogenetic evaluation and web-based resources, revealed that genotype B predominated (73.83%, 95% CI: 64.59–81.36), with genotype C comprising 26.17% (95% CI: 18.64–35.40). At the sub-genotype level, B4 was the most frequent (64.49%, 95% CI: 54.88–73.04), followed by C1 (14.95%, 95% CI: 9.32–23.13), B2 (9.35%, 95% CI: 5.07–16.60) and C2 (6, 95% CI: 3.12–13.19). Rarer sub-genotypes included C3 (~0.93%) and C5 (3.74%, 95% CI: 1.39–9.64). To explore whether genotype and sub-genotype distributions differed by infectious status, we next compared the HBsAg-positivity and OBI groups. Figure 3 shows the distribution of HBV genotypes and sub-genotypes by group (HBsAg-positivity, OBI).

Across the OBI and HBsAg-positivity groups, the distribution of genotypes and sub-genotypes did not differ significantly (Fisher’s exact *p* = 0.082). In logistic regression treating OBI vs. HBsAg-positivity as the outcome, sub-genotype was not associated with group status (Wald χ^2^ = 1.15, *p* = 0.77). Several rare sub-genotypes produced empty cells/perfect prediction and were omitted. These findings indicate no robust evidence for sub-genotype significant differences between OBI and HBsAg-positivity in our cohort.

### 3.6. Surface Gene Mutations Associated with Occult HBV Infection

Sequencing the entire Pre-S1/Pre-S2/S region was successfully performed for all 107 viremic samples. Analysis of the Pre-S1 and Pre-S2 regions revealed multiple polymorphisms. However, no clinically significant or previously described mutations associated with hepatocarcinogenesis (e.g., start codon mutations, large deletions) were identified in our cohort. Additionally, no mutations in the RT region overlapping the S gene predicted to affect HBsAg expression or serological clearance were identified.

We observed a diverse spectrum of amino acid substitutions in the HBsAg major hydrophilic region (MHR, aa 99–169, encompassing the “a” determinant, aa 124–147). Table 5 summarizes all detected variants and their frequencies.

Table 6 summarizes the distribution of major HBsAg escape substitutions within the MHR (“a” determinant) in HBsAg-positivity (HBV DNA^+^/HBsAg^+^, *n* = 70) versus OBI (HBV DNA^+^/HBsAg^−^, *n* = 37).

Overall, several canonical escape changes were significantly enriched in OBI, yet absent or rare in HBsAg-positivity. These included T116N/A/I/S, Q129H/G, M133L/T/V/I, K141R/T/E/Q, P142H/R, D144A/H, and G145R/A (all *p* < 0.05 by two-sided Fisher’s exact test. Effect sizes were large, with odds ratios (ORs) estimated using the Haldane-Anscombe 0.5 continuity correction and 95% CIs from the log-OR (e.g., OR = 34.67 for T116, OR = 47.00 for K141, OR = 40.63 for G145). In contrast, changes such as T126I/N/S and M133L/T/V/I did not differ significantly between groups, and P127T trended lower in OBI.

In addition, a group comparison (OBI, HBsAg-positivity) of per-patient burden of major HBsAg escape substitutions was conducted using a two-sided Wilcoxon rank-sum (Mann–Whitney) test. OBI featured a significantly higher number of escape mutations per individual than HBsAg-positivity (z = −5.344, *p* < 0.001, exact *p* < 0.001). Rank sums were lower than expected in HBsAg-positivity (observed 3028 vs. expected 3780) and higher than expected in OBI (2750 vs. 1998). Thus, OBI patients carry a significantly higher mutation burden than HBsAg-positivity.

**Figure 4:**
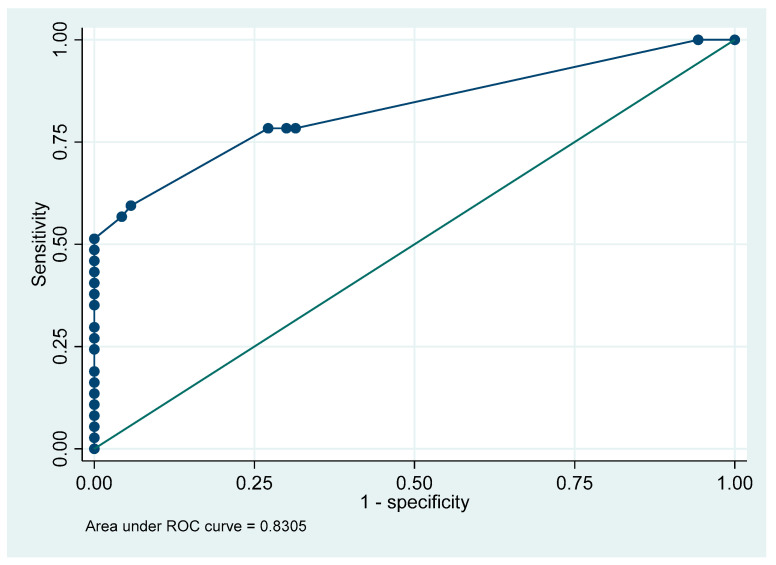
ROC curve of a LASSO logistic model predicting HBsAg negativity from escape mutations. The *x*-axis shows “1−specificity” (proportion of misclassifying HBsAg-positive samples). The *y*-axis shows sensitivity (proportion of correctly identifying HBsAg-negative samples). Each dot corresponds to a different probability threshold of the model. The 45° diagonal represents random guessing (AUC = 0.5). Curves that bow farther toward the top-left corner indicate better discrimination.

To further investigate the role of these mutations in HBsAg negativity, a penalized logistic regression model (LASSO) was developed to assess whether a specific panel of escape mutations could predict OBI. The curve plots the model’s ability to correctly identify true HBsAg-negative samples (sensitivity, *y*-axis) against its rate of incorrectly classifying true HBsAg-positive samples as negative (false positive rate, *x*-axis). The model demonstrated good discriminatory ability to distinguish between HBsAg-negative (OBI) and HBsAg-positive individuals based on their mutational profiles. The receiver operating characteristic (ROC) curve analysis confirmed the model’s strong performance, yielding an area under the curve (AUC) of 0.83 (95% CI: 0.75–0.92). This indicates that the combination of these escape mutations carries significant predictive information for occult HBV status (Figure 4).

There was no significant difference in the prevalence of major escape mutations by gender or across age groups. When analyzing the distribution of escape mutations by genotype and sub-genotype, only the T126I/N/S substitution showed a statistically significant difference. This mutation was strongly associated with genotype C, occurring in 89.29% of genotype C isolates, compared to just 2.53% of genotype B isolates (OR = 225, *p* < 0.001). This pattern was even more pronounced at the sub-genotype level, where T126I/N/S was universally present in all C2 isolates (100%), while being completely absent from B2 isolates (0%). Furthermore, its prevalence was also significantly higher in sub-genotype C5 (50%) compared to B4 (2.90%) (OR = 27.03, *p* = 0.013).

## 4. Discussion

This study provides an integrated serological and virological assessment of hepatitis B virus infection in an apparently healthy adult population in Southern Vietnam. Our findings are consistent with known epidemiological patterns in this hyperendemic setting and further characterize the burden of both overt and occult HBV infection. By combining sensitive HBsAg and HBV DNA testing with surface gene sequence analysis, this study provides additional molecular context to the observed serological profiles and helps to further characterize viral features associated with different HBV infection phenotypes.

The observed prevalence of HBsAg positivity (17.6%) confirms the burden of HBV infection in Southern Vietnam remains high, particularly among adults born before the introduction of universal infant vaccination since the 1990s. This figure exceeds the 7.20% estimated for Vietnam’s general population by the WHO (2022) and the 10.62% pooled prevalence from recent systematic reviews on Vietnam [5,24], but is comparable to rates reported in other Vietnamese studies conducted in similar healthcare-based settings, including cohorts from Thai Binh (19.0%), Binh Thuan (15.3%), and Ho Chi Minh City (18.8%) [38,39,40].

The overall serological profile of the cohort reflects intense cumulative exposure and declining immunity. The high prevalence of anti-HBc IgG (63.7%), consistent with reports from Vietnam and neighboring Southeast Asian countries [38,39,40,41,42], contrasts with the relatively low prevalence of protective anti-HBs IgG (38.5%). This imbalance is manifested by the predominance of the ‘anti-HBc IgG only’ profile (31.5%), a pattern characteristic of populations with high historical exposure and waning immune protection. Although higher than in some Vietnamese cohorts [40], similarly high levels have been described not only in endemic regions (e.g., China), but even in intermediate or low-prevalence settings (Korea, Europe, U.S.) [41,42,43,44].

Age and gender were key modifiers of these patterns. Markers of HBV infection and exposure increased with age and were more frequent in men, whereas anti-HBs IgG declined after the age of 30. The male predominance in HBsAg positivity is consistent with global epidemiological observations [45,46,47]. The age-related shift toward higher HBsAg and anti-HBc IgG prevalence, accompanied by declining anti-HBs IgG, is compatible with a vaccination cohort effect following the introduction of infant immunization programs [24], as well as gradual antibody loss over time [6,48]. Integrated age-based analyses (Table 3) capture these opposing trends and provide an explanation for the high prevalence of overt infection and the ‘anti-HBc IgG only’ profile among individuals over 30 years of age, highlighting persistent gaps in adult immunity despite vaccination success in younger generations.

Another key finding of our study is the pronounced gender disparity in HBsAg-positivity, with males having nearly three-fold higher odds of being positive for both HBV DNA and HBsAg compared to females (OR 2.89, *p* < 0.001). These data are strongly corroborated by our previous study on Vietnamese blood donors, which reported a near-identical risk for overall HBV viremia (RR = 2.95) [14]. This male predominance in CHB is a globally recognized phenomenon, widely attributed to a combination of factors, including sex hormones (where androgens may impair viral clearance) and differences in immune responses between genders [45,46]. Interestingly, this striking disparity completely disappears in the context of OBI. Our analysis shows no significant difference in the odds of OBI between males and females, a finding supported by other regional studies that also reported a lack of gender association [49].

Our study identifies a substantial prevalence of OBI at 9.32%. This estimate contributes additional data on the burden of OBI in a healthy, non–blood donor adult population in Vietnam, for which local evidence remains limited. Notably, this prevalence aligns closely with our recent findings in Southern Vietnamese blood donors (8.6%) [14], a cohort with a comparable age distribution analyzed using the same high-sensitivity PCR methodology. Furthermore, our data align perfectly with regional meta-analyses that have estimated the pooled OBI prevalence to be between 9% and 10.5% for the general population of Southeast Asia [50,51]. In contrast, our OBI percentage is substantially higher than the 0.3% reported by Tung et al. in a cohort of highly screened blood donors from Northern Vietnam [52], highlighting the strong influence of recruitment criteria and diagnostic sensitivity. Differences in reported OBI prevalence may reflect a range of additional factors, including regional variations in HBV epidemiology. Inter-individual differences in host immune control, viral genotype distribution, and the occurrence of surface gene variants that may reduce HBsAg detectability play a role [8,11,16,53]. Moreover, heterogeneity in historical exposure patterns, vaccination coverage, and the cumulative effects of immune pressure over time might also contribute to variability in the size of the occult reservoir [6,24,48]. Collectively, these considerations indicate that observed OBI prevalence likely reflects a complex interplay of biological, epidemiological, and methodological influences.

These findings indicate that while the hidden reservoir of OBI remains stable, the visible burden of HBsAg-positivity, indicating potential chronic HBV infection, is greater in the general population than in blood donors. This study adds to the limited body of evidence assessing HBV viral burden in adults undergoing routine health examinations in Vietnam. Many previous epidemiological surveys were limited to seroprevalence data [38,39,40,54]. In addition, other local studies employing HBV DNA testing have mainly targeted specific subgroups, such as HBsAg-negative blood donors, rather than providing a comprehensive prevalence profile. The measured prevalence of OBI is strongly influenced by the analytical sensitivity of the diagnostic assays employed. Highly sensitive HBsAg tests can lower apparent OBI numbers by reclassifying borderline cases as overt infections, whereas more sensitive nucleic acid tests (NAT) increase the numbers by detecting low-level viremia, leading to significant between-study variability [8,53].

In this study, we addressed this challenge by pairing a high-sensitivity HBsAg assay (LoD = 0.01 IU/mL) with a low-detection-limit NAT (5 IU/mL). Specimens with detectable HBV DNA underwent additional extraction from ≥5 mL plasma to obtain sufficient material for sequencing. This approach was designed to minimize the misclassification of low-level chronic infections, while maximizing the detection of true low-level viremia, thereby providing a more accurate and biologically plausible estimate of the OBI burden. This rigorous methodology likely explains why our reported OBI prevalence is higher than that in some previous reports from Vietnam which may have utilized assays with higher detection limits (i.e., lower sensitivity).

This study moves beyond prevalence to elucidate mutation accumulation underlying the OBI phenotype. Our data provide insight into the genetic characteristics of HBV circulating within a cohort of healthy Vietnamese adults, particularly those contributing to the significant burden of OBI. In Vietnam, data investigating MHR variation in healthy or low-acuity cohorts remain limited. We demonstrate conclusively that individuals with OBI carry a significantly higher burden of S gene escape mutations compared to those with chronic hepatitis B (*p* < 0.001). This was not merely a quantitative difference. A clear pattern of specific, clinically significant escape variants was substantially enriched in, or exclusively detected within, the OBI group. These include T116N/A/I/S, Q129H/G, M133L/T/V/I, K141R/T/E/Q, P142H/R, D144A/H, and the classic G145R/A. This presents a stark contrast to previous Vietnamese studies on CHB/HCC cohorts, which reported escape mutants at very low frequencies of 0.7–2.2% [17,55]

This strong association provides powerful evidence for the hypothesis that the accumulation of mutations within the MHR, particularly the “a” determinant, is a primary molecular mechanism driving HBsAg negativity in OBI [56]. The most prominent examples of this enrichment include the G145R mutation (21.62% in OBI vs. 0% in HBsAg-positivity), K141R/T/E/Q (24.32% in OBI vs. 0% in HBsAg-positivity), and T116N/A/I/S (18.92% in OBI vs. 0% in HBsAg-positivity). These mutations are known to impair the synthesis and secretion of HBsAg from hepatocytes, causing its concentration in the blood to fall below detectable levels, although the virus continues to replicate [56,57,58]. In addition, it is important to acknowledge that HBsAg negativity in these cases may result not only from impaired secretion or low viral load, but also from diagnostic escape, wherein the conformational changes induced by these mutations prevent detection by the specific monoclonal antibodies used in standard commercial assays.

Crucially, the impact of these MHR mutations extends beyond diagnostics insofar as they are well-characterized vaccine escape variants. Mutations such as G145R alter the conformational structure of the “a” determinant, the primary target for neutralizing Abs induced by current second-generation vaccines. This structural change can significantly reduce antibody binding, potentially allowing the virus to infect vaccinated individuals [59,60]. Therefore, our finding that the OBI reservoir is enriched with these variants highlights the circulation of a viral population that poses a direct threat to the effectiveness of current immunization programs. This underscores the urgent need to not only enhance molecular surveillance, but also to accelerate the development and implementation of third-generation vaccines (containing Pre-S1/Pre-S2 antigens) and mRNA-based platforms designed to provide broader protection against such escape mutants [60].

In this study, the LASSO method was employed as the statistical tool for its dual capability of building a predictive model, while simultaneously performing variable selection from high-dimensional data. The suitability of LASSO-based methods for analyzing complex genetic data is well-established [61]. The robustness of this “mutational signature” was confirmed by our LASSO regression model. It predicted OBI status with high accuracy (AUC = 0.83) based solely on the mutational profile, confirming that these mutations carry significant predictive information. The enrichment of these mutations in the OBI group is likely the result of long-term immunological pressure from the host, which favors the survival of viral variants that can evade immune recognition [62,63]. To the best of our knowledge, no prior study has applied LASSO logistic regression to build an OBI classifier using only the S gene mutational profile. Prior works on OBI primarily reported mutation frequencies or functional impacts without penalized ML modeling. Similarly, Jia et al. effectively used LASSO to create a powerful predictive model for HCC by analyzing quasispecies diversity in the PreS region, successfully identifying mutations critical for the classification of HCC and CHB patients [64].

In the cohort of conditionally healthy Vietnamese adults with detectable HBV DNA, genotype B predominated (73.8%), driven by sub-genotype B4 (64.5%) with a smaller contribution from sub-genotype B2 (9.35%). Genotype C (26.2%) was represented mainly by C1 and C2, alongside rarer C3/C5 lineages. This finding aligns with the established genotypic landscape in Vietnam. Genotypes B/C are consistently reported as the most prevalent (genotype B predominant), especially sub-genotypes B4 and C1 [14,18,19,38,65,66,67]. Regionally, healthy population surveys in mainland Southeast Asia identify a similar B/C co-dominance, but with notable country-specific variations. For instance, in Myanmar’s general population, genotype C (primarily C1) is the leading lineage, whereas a B4 majority has been reported in Laos and parts of southern Vietnam, which is consistent with our findings [68,69]. Together, these data confirm a B4-centric genotypic structure in healthy Vietnamese adults, with a substantive secondary contribution from C1/C2.

A noticeable trend was observed for genotypic composition by clinical status. Genotype B (especially B4) accounted for a larger share of HBsAg-positive chronic infection, while genotype C contributed a relatively greater fraction to OBI. However, this distributional difference did not reach statistical significance. We also identified distinct genotype-specific mutational patterns. The T126I/N/S escape mutation exhibited a strong and exclusive association with genotype C. This has also been reported in Vietnamese HCC patients, suggesting this is an intrinsic, genotype-linked antigenic signature rather than a disease-stage artifact [18].

This study has several methodological strengths. It provides an integrated analysis of serological, virological, and genetic features of HBV infection within a single cohort of apparently healthy adults in Vietnam, an approach that has been relatively limited in previous studies. The use of highly sensitive HBsAg (0.01 IU/mL) and HBV DNA assays (≥5 IU/mL), together with large-volume plasma extraction, likely improved detection of low-level viremia and enabled sequencing of samples that might otherwise have been missed by less sensitive methods. This enhanced our ability to detect and successfully sequence low-viremic cases that are often missed by conventional methods. This may explain the higher frequency of escape mutations compared to other reports. In addition, the application of a LASSO-based regression approach allowed the identification of a set of surface gene mutations associated with HBsAg negativity, providing a data-driven framework to explore mutational patterns linked to occult infection.

The study also has limitations. The multi-center, cross-sectional design recruited adults from routine health examination settings. However, the small number of participants recruited from each individual center may limit the generalizability of the findings or introduce bias related to local demographics, health-seeking behavior, or socioeconomic factors. Our study population was recruited from several healthcare facilities in Southern Vietnam. As such, the reported prevalence values reflect this specific region, and they may not be generalizable to the entire country. HBsAg positivity reflects current antigen detection; it does not confirm chronic infection, which requires evidence of persistence for ≥6 months. Individual vaccination histories were unavailable, and the absence of anti-HBc IgM testing limited the classification of atypical serological profiles. As individual vaccination histories were not available, classification of anti-HBs origin was based on serological profiles rather than documented immunization records. Finally, familial or household clustering could not be assessed, although the high genetic heterogeneity of circulating HBV strains supports cohort representativeness.

## 5. Conclusions

In conclusion, our study reveals a significant burden of both chronic and occult HBV infection within a healthy adult Vietnamese population, concentrated primarily in older, pre-vaccination era cohorts. Critically, we establish a definitive molecular link between the occult phenotype and a higher burden of S gene escape mutations. This finding is critical, revealing a substantial viral reservoir of escape variants that not only evades detection by conventional HBsAg screening, but also threatens the neutralizing efficacy of vaccine-induced antibodies.

Our findings have critical implications for HBV control strategies. For diagnostics, we recommend upgrading national testing algorithms to incorporate anti-HBc and HBV DNA alongside HBsAg in high-risk settings. This should be coupled with the use of high-sensitivity assays for all serological markers and nucleic acid tests (NAT). For prevention, immunization policy should continue to prioritize a timely birth dose while addressing adult immunity gaps through targeted catch-up vaccination. Furthermore, to counter the threat posed by escape variants, it is imperative to accelerate the development of next-generation vaccines, such as those incorporating pre-S1/pre-S2 antigens or utilizing mRNA platforms. Finally, additional nationwide, longitudinal studies are warranted to fully elucidate the complex dynamics between serological markers, the evolution of circulating viral variants, and diagnostic assay performance.

## Figures and Tables

**Table 1:** Prevalence of HBV serological profiles among the study population (N = 397).

HBV Serological Profile			*n*	Percentage (95% CI)
HBsAg	Anti-HBs IgG	Anti-HBc IgG		
−	−	−	64	16.12% (12.49–19.75)
−	+	+	68	17.13% (13.41–20.85)
−	+	−	70	17.63% (13.87–21.40)
−	−	+	125	31.49% (26.90–36.07)
+	−	−	9	2.23% (0.80–3.74)
+	+	−	1	0.25% (0.00–0.74)
+	−	+	46	11.59% (8.42–14.75)
+	+	+	14	3.52% (91.70–5.35)

**Table 2:** Prevalence of viremic HBsAg-positive status and occult HBV infection by gender.

Gender	OBI Among Healthy Adults		HBV DNA^+^, HBsAg^+^ (N = 70)		HBV DNA^+^, HBsAg^−^ (N = 37)	
	***n***	**% (95% CI)**	***n***	**% (95% CI)**	***n***	**% (95% CI)**
Male	195	9.23% (5.56–14.20)	49	70% (57.87–80.38)	18	48.65% (31.92–65.60)
Female	202	9.41% (5.76–14.30)	21	30.0% (19.62–42.13)	19	51.35% (34.40–68.08)

**Table 3:** Adjusted age-trend odds ratios for HBV markers among conditionally healthy individuals.

HBV Marker	Adjusted Odds Ratio per ^†^ Step	Adjusted Lower 95% CI	Adjusted Upper 95% CI	Adjusted *p*-Value
anti-HBs IgG^+^	0.22	0.16	0.31	<0.001
anti-HBc IgG^+^	1.37	1.12	1.66	0.0021
HBsAg^+^	1.74	1.37	2.21	<0.001
anti-HBc IgG^+^, anti-HBs IgG^+^	0.42	0.30	0.58	<0.001
Anti-HBc IgG^+^ only	1.55	1.28	1.89	<0.001
Anti-HBs IgG^+^ only	0.28	0.19	0.42	<0.001
HBV DNA^+^	1.59	1.30	1.96	<0.001
HBV DNA^+^, HBsAg^+^	1.74	1.37	2.21	<0.001

Notes. Age coded ordinally as five categories: <30, 30–39, 40–49, 50–59, ≥60 years. **^†^** OR values reflect change per one-step increase in age-group.

**Table 4:** Serological profile prevalence in conditionally healthy HBV DNA^+^ individuals (N = 107).

HBV Serological Profile		HBsAg-Positivity (HBV DNA^+^, HBsAg^+^) *n* = 70		OBI (HBV DNA^+^, HBsAg^−^) *n* = 37	
Anti-HBs IgG	Anti-HBc IgG	***n***	% (95% CI)	***n***	% (95% CI)
−	−	9	12.86% (6.75–23.12%)	2	5.41% (1.29–19.97)
+	+	14	20.00% (11.39–31.27%)	12	32.43% (19.06–49.46)
+	−	1	1.43% (0.19–9.75%)	4	10.81% (4.0–26.18)
−	+	46	65.71%, (53.70–76.0%)	19	51.35% (35.14–67.29)

**Table 5:** Identified MHR mutations (aa 99–169).

Mutation	Frequency of Occurrence in the Group (N = 107)			
	*n*	%	95% CI−	95% CI+
Y100C	1	0.93	0.17	5.10
L109P/R	2	1.87	0.51	6.56
I110L	28	26.17	18.77	35.22
P111A/R	2	1.87	0.51	6.56
G112A/R	2	1.87	0.51	6.56
T113S/A/L	77	71.96	62.80	79.60
T114S/A/R	106	99.07	94.90	99.83
T116N/A/I/S	7	6.54	3.20	12.89
S117R/T	2	1.87	0.51	6.56
T118S/A	3	2.80	0.96	7.92
G119A/R	4	3.74	1.46	9.22
P120S/T/R	7	6.54	3.20	12.89
C121S/W	5	4.67	2.01	10.48
K122R/G	69	64.49	55.06	72.91
C124W	3	2.80	0.96	7.92
T125P/S	2	1.87	0.51	6.56
T126I/N/S	27	25.23	17.96	34.22
P127T	6	5.61	2.60	11.70
Q129H/G	4	3.74	1.46	9.22
N131T/I	107	100.00	96.53	100.00
S132A/F	2	1.87	0.51	6.56
M133L/T/V/I	13	12.15	7.24	19.68
F134L	2	1.87	0.51	6.56
P135R	1	0.93	0.17	5.10
S136A/L	2	1.87	0.51	6.56
C137S	1	0.93	0.17	5.10
C138S/W	2	1.87	0.51	6.56
C139S/W	4	3.74	1.46	9.22
K141R/T/E/Q	9	8.41	4.49	15.22
P142H/R	3	2.80	0.96	7.92
S143T/M/A/W	80	74.77	65.78	82.04
D144A/H	3	2.80	0.96	7.92
G145R/A	8	7.48	3.84	14.06
N146K	1	0.93	0.17	5.10
C147R/W	3	2.80	0.96	7.92
A159V	1	0.93	0.17	5.10
K160R	25	23.36	16.36	32.22
Y161F/S	37	34.58	26.24	43.98
E164G	1	0.93	0.17	5.10

**Table 6:** Distribution of major escape mutations among conditionally healthy individuals with HBsAg-positive or OBI status.

Mutation	Frequency of Occurrence in the HBsAg-Positivity Group (N = 70)		Frequency of Occurrence in the OBI Group (N = 37)		OBI vs. HBsAg-Positivity (*Two-Sided Fisher’s Exact Test)*	
	*n*	% (95% CI)	*n*	% (95% CI)	OR	*p*-Value
T116N/A/I/S	0	0%	7	18.92% (9.48–34.20)	34.67	0.0004
P120S/T/R	2	2.86% (0.79–9.83)	5	13.51% (5.91–27.98)	4.64	0.0470
T126I/N/S	16	22.86% (14.59–33.95)	11	29.73% (17.49–45.78)	1.43	0.4866
P127T	5	7.14% (3.09–15.66)	1	2.70% (0.48–13.82)	0.49	0.6621
Q129H/G	0	0%	4	10.81% (4.29–24.71)	18.94	0.0128
M133L/T/V/I	4	5.71% (2.24–13.79)	9	24.32% (13.36–40.12)	4.93	0.0101
F134L	0	0%	2	5.41% (1.50–17.70)	9.93	0.1174
C139S/W	1	1.43% (0.25–7.66)	3	8.11% (2.80–21.30)	4.70	0.1182
K141R/T/E/Q	0	0%	9	24.32% (13.36–40.12)	47.00	0.0000
P142H/R	0	0%	3	8.11% (2.80–21.30)	14.30	0.0391
D144A/H	0	0%	3	8.11% (2.80–21.30)	14.30	0.0391
G145R/A	0	0%	8	21.62% (11.39–37.20)	40.63	0.0001

## Data Availability

The raw data supporting the conclusions of this article will be made available by the authors on request.
